# Morphology and Structure of Electrodeposited Prussian Blue and Prussian White Thin Films

**DOI:** 10.3390/ma12071103

**Published:** 2019-04-03

**Authors:** Bruna F. Baggio, Cristiano Vicente, Silvia Pelegrini, Cristiani Campos Plá Cid, Iuri Stefani Brandt, Milton André Tumelero, André A. Pasa

**Affiliations:** 1 Laboratório de Filmes Finos e Superfícies, Universidade Federal de Santa Catarina, 88.040-900 Florianópolis, Brazil; bfbaggio@gmail.com (B.F.B.); vicentecristiano@gmail.com (C.V.); pelegrini.silvia@gmail.com (S.P.); cristiani.campos@ufsc.br (C.C.P.C.); iuribrandt@gmail.com (I.S.B.); 2 Laboratório de Resistividade, Magnetismo e Supercondutividade, Instituto de Física, UFRGS, 90040-060 Porto Alegre, Brazil; matumelero@if.ufrgs.br

**Keywords:** Prussian Blue, Prussian White, Electrodeposition, Hexacyanoferrate

## Abstract

The compound Prussian Blue (PB), and its reduced form Prussian White (PW) are nowadays considered, in applied and fundamental research groups, as potential materials for sustainable energy storage devices. In this work, these compounds were prepared by potentiostatic electrochemical synthesis, by using different deposition voltages and thicknesses. Thick, compact and uniform layers were characterized by scanning electron microscopy, X-ray diffraction, and Raman spectroscopy. Results have shown a well-defined transition voltage for growing Prussian Blue phases and a strong dependence of the morphology/growing orientation of the samples as a function of applied potential and thickness. For the negative potential tested of −0.10 V vs. SCE, a mixture of cubic and rhombohedral phases was observed.

## 1. Introduction

The crystal structure of Prussian Blue (PB) was described by Keggin and Miles [1] as a cubic array with edges formed by Fe^2+^–C≡N–Fe^3+^ chains, with six cyano groups linked to each Fe ion, corresponding to a cubic face-centered unit cell with a lattice parameter 10.2 Å. The chemical forms (PB and analogs) associated to the metal–organic open framework of Prussian Blue are described by the general formula $A_{x}M_{y}^{a}{\left[M^{b}{\left(CN\right)}_{6}\right]}_{z}$, where A is an alkaline metal, M a transition metal, *a* and *b* oxidation numbers and *x*, *y*, and *z* stoichiometric numbers. Prussian Blue has two phases, ${\mathrm{Fe}}_{4}^{3+}{\left[{\mathrm{Fe}}^{2+}{\left(CN\right)}_{6}\right]}_{3}$ and ${\mathrm{KFe}}^{3+}\left[{\mathrm{Fe}}^{2+}{\left(\mathrm{CN}\right)}_{6}\right]$ and its reduced forms $K_{4}{\mathrm{Fe}}_{4}^{2+}{\left[{\mathrm{Fe}}^{2+}{\left(\mathrm{CN}\right)}_{6}\right]}_{3}$ and $K_{2}{\mathrm{Fe}}^{2+}\left[{\mathrm{Fe}}^{2+}{\left(\mathrm{CN}\right)}_{6}\right]$, that are known as Prussian White (PW). The PB phase with the alkali metal is usually called as soluble and the one without as insoluble [2], with the alkali metal occupying the cubic cavities of the Fe–C≡N–Fe network.

Prussian Blue materials are relevant for several applications such as electrochromic systems [3], electrochemical sensors [4,5], and energy storage devices [6,7,8,9]. Prussian Blue and analogs are also potential materials for aqueous electrolyte and sodium ion batteries [6,10,11,12,13,14,15]. These materials are usually prepared by chemical synthesis by precipitation methods [8,10,12,15,16,17,18,19,20,21]. Electrodeposition is an alternative technique for growing Prussian Blue and analogs, adequate for producing films with high reproducibility and low cost. Systematic studies of electrodeposition of Prussian Blue and White, as a function of applied potential and thickness, for example, are not found in the literature. Intense work was devoted in the past for understanding the potentiostatic deposition of PB and intercalation of K for the stabilization of the material in the soluble phase [22,23,24,25,26]. Electrodeposited samples of Prussian Blue and analogs were also tested with success as electrodes for batteries [27,28,29,30,31,32,33,34].

Although the literature about hexacyanoferrates (Prussian Blue and analogs) is extensive, it is still lacking a description of the properties of the Prussian Blue deposits when prepared by electrochemical synthesis, i.e., using potentiostatic deposition. In the present work, we obtained thick and compact thin films of both phases, Prussian Blue and Prussian White, that are suitable to be used as electrodes in applications such as supercapacitors and batteries. At the same time, we demonstrated the influence of electrodeposition potential and thickness on the structure and morphology of PB and PW layers, and the deposition voltage where the transition from PB to PW occurs.

## 2. Experimental Section

The electrochemical deposition, potentiostatic mode, was performed at room temperature with Au/Si substrates as working electrodes, a Pt foil as counter-electrode, and saturated calomel electrode (SCE) as reference. All the voltages in the text refer to this reference electrode. The working electrodes were prepared by evaporating 50 nm Au on 5 nm Cr on Si (100) substrates with sizes of 1cm × 1cm at a pressure of 10^−7^ Torr. The deposits occurred in a circular area of 0.5 cm^2^ on the surface of the working electrode, delimited by an adhesive tape. The electrolyte for the electrochemical synthesis consisted of 0.25 mM K_3_Fe(CN)_6_, 0.25 mM FeCl_3_, 1.0 M KCl and 5.0 mM HCl [35], with a pH of 2.2. PB and PW layer formation was promoted by applying constant potential values in the interval from −0.2 to 0.5 V, with different electrochemical charge values, using an Autolab PGSTAT 302N electrochemical workstation, MetrohmAG, Herisau, Switzerland.

The morphologies of deposits were investigated using a JEOL JSM-6701F field emission scanning electron microscope (FEG-SEM JSM-6701F JEOL, Tokyo, Japan) at 5 kV. The thickness of the samples was measured with a Dektak XT profilometer from Bruker Company, Billerica, USA and compared with results obtained from FEG-SEM cross-section images. This procedure was done in many samples to check the results obtained by both techniques and to confirm the good reproducibility of the deposition process. Structural properties were studied by XRD (X-ray diffractometry) (X-Ray Diffractometer model Xpert PRO MPD, Panalytical, Almelo, Netherlands, with CuKα_1_ radiation, 1.541 Å) and Raman spectroscopy (Renishaw 2000 micro-Raman spectrometer, Gloucestershire, UK). Raman spectra were obtained using an excitation wavelength of 514.5 nm, power set at 20 mW and spectral resolution of 0.33 cm^−1^. In order to increase the signal-to-noise ratio, ten spectra were obtained for each sample (with an acquisition time of 10 s) and an average spectrum was obtained.

## 3. Results and Discussions

**Figure 1:**
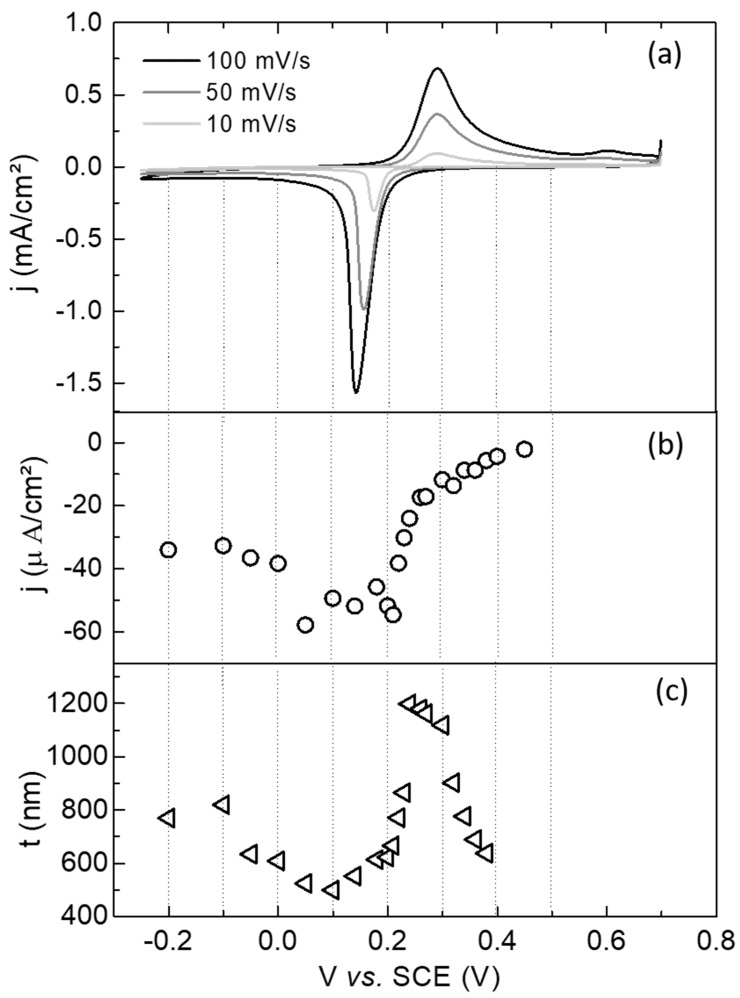
(**a**) Voltammetric curves at different scanning rates (10, 50, 100 mV/s) on Au/Si substrates. (**b**) Current densities at saturation and (**c**) thicknesses as a function of the applied constant potential for growing the layers.

Figure 1a shows typical voltammograms at different scanning rates for the voltage ranging from −0.25 to 0.70 V. The reduction peak is associated with the PW growth with simultaneous intercalation of K and the oxidation peak at ~0.30 V is due to the deintercalation of K and consequent formation of PB [35]. Figure 1b shows the current density values obtained from current transients in the saturation region (long deposition times) for applied constant potentials in the range from −0.2 to 0.50 V. Figure 1c shows the dependency of the thickness of the deposits as a function of deposition potential in the same range of voltages. The depositions were stopped when the electrochemical charge hit 50 mC. As a general trend, deposits grown at more positive potentials than 0.20 V presented smaller current densities (in module) and were associated with the growth of PB, as was described above from the voltammetric curves in Figure 1a. For potentials more negative than about 0.20 V, the growth of PW layers is expected. Concerning the thickness, layers grown in the potential range of 0.22 to 0.30 V were a factor 2 thicker than samples grown in the interval from 0.20 to −0.05 V. As the electrodeposited charge is the same for these layers, the difference in the thickness is due to the fact that for PB formation only one electron is needed to reduce the Fe^3+^ ions while for PW formation two electrons are needed to reduce Fe^3+^ and [Fe(CN)_6_]^3−^ species.

**Figure 2:**
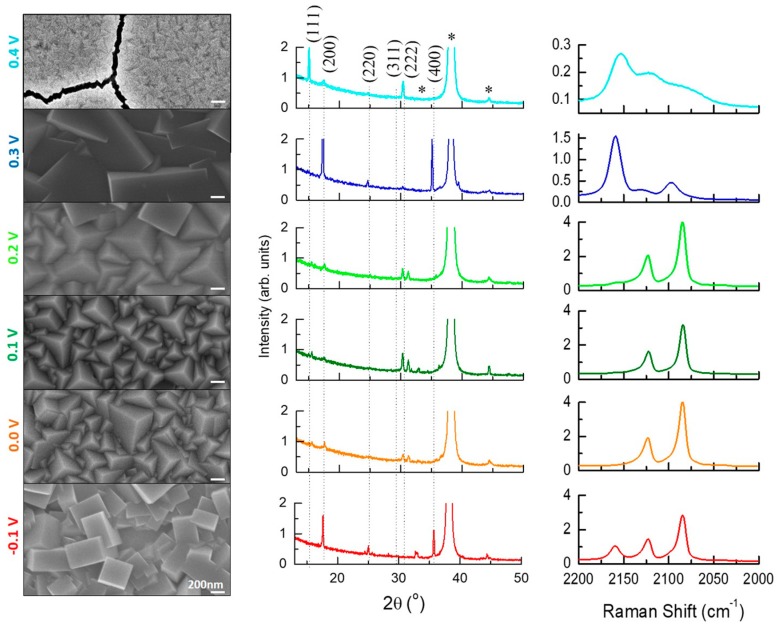
FEG–SEM images (column 1), XRD diffractograms (column 2), and Raman spectra (column 3) of thin films electrodeposited with 50 mC at 0.40, 0.30, 0.20, 0.10, 0.00, and −0.10 V (from top to bottom). The peaks indicated by (*) are from the Au substrate. The dotted lines correspond to peak positions for the PB structure as obtained from the ICSD database code: 23102.

In Figure 2, column 1 presents SEM–FEG images of films deposited at 0.40, 0.30, 0.20, 0.10, 0.00, and −0.10 V, from top to bottom, with a charge of 50 mC. In order to describe the structural evolution of the samples at different deposition potential conditions, X-ray diffractograms and Raman spectra were obtained from these samples and are presented in columns 2 and 3. The sequence of SEM images in column 1 shows that, for the most positive potential, small pyramidal grains are formed (less than 200 nm in lateral size). The formation of cracks also occurred, as previously observed for these relatively high voltages [26]. From 0.30 to −0.10 V, large grains are grown and the morphology that starts as cubes evolves to pyramids and returns back to cubes with decreasing voltage. Following this morphology with X-rays, we can see in column 2 that for the deposition potential of 0.40 V the growth of pyramids is predominant, i.e., <111> out-of-plane orientation, but other reflections as (200) and (311) are also present in the diffractogram that indicates the presence of crystals with cubic and inclined pyramids morphology, respectively. In the sequence of tested potentials, we observed that the film deposited at 0.30 V has a <100> preferential growth direction in agreement with the cubes morphology at the surface, i.e., intense reflections associated with (200) and (400) planes are observed. Lower values of deposition potentials, from 0.20 to 0.00 V, resulted in the preferential growth of the <311> direction with some crystals in the <111>, also in agreement with SEM images. Finally, the film grown at the negative applied potential of −0.10 V had a predominantly cubic morphology with <100> preferential orientation, again in concordance with the corresponding SEM image in column 1. For this sample and the sample prepared at 0.00 V, it was possible to observe peaks with low intensity at 2θ~25^°^ that are associated with the rhombohedral phase [19,36]. This phase could be responsible for the change in morphology of the sample prepared at −0.10 V, where the rhombohedral peak is more intense.

In column 3 of Figure 2, the structural results obtained by Raman spectroscopy are presented. The Raman spectrum for hexacyanoferrates exhibits CN stretching bands, ν(CN), between 2200 and 2000 cm^−1^. The ν(CN) bands observed in the spectrum obtained for PB films grown at 0.40 V exhibited three broad cyanide vibration peaks at about 2160, 2120, and 2080 cm^−1^. The broadness of the peaks indicates strain in the layers [37], in agreement with the cracks observed by SEM. In the spectrum of the sample prepared at 0.30 V, the vibration bands appear more defined, consistent with the large crystals observed on the surface by SEM, with peaks at 2160, 2122, and 2090 cm^−1^. For deposits with the voltage in the interval from 0.20 to −0.10 V, the stretching mode at 2160 cm^−1^ is much less intense or even disappears. Since the wavenumber peaks at ~2210 and 2090 cm^−1^ can be associated with the cyanide group attached to Fe^2+^, and the 2160 cm^−1^ peak with Fe^3+^ íons [38,39,40,41], the disappearance of this last peak by using more negative deposition potentials promotes the reduction of Fe^3+^ species to Fe^2+^ and the consequent formation of PW, as expected, which was also observed by other authors [8,9,42,43].

**Figure 3:**
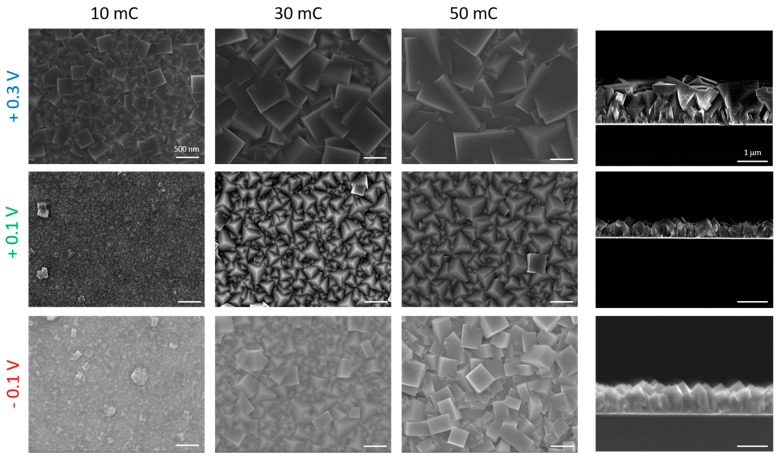
Columns 1, 2 and 3 show SEM–FEG top-view images of samples with an electrodeposited charge of 10, 30 and 50 mC, respectively. The lines 1, 2 and 3 are for deposition potentials of 0.30, 0.10 and −0.10 V, respectively. Column 4 is cross-section SEM–FEG images of samples grown with 50 mC at the three different potentials used.

Figure 3 shows SEM–FEG images for samples prepared using different deposition potentials and charges. SEM images are presented in column 1, 2 and 3 for 10, 30 and 50 mC, respectively. The deposition potentials are 0.30 V in the first line, 0.10 V in the second and −0.10 V in the third one. An increase in the grain size can be observed for all samples as the film grows, i.e., as a function of reduced charge (thickness). Films deposited at 0.30 and 0.10 V presented predominantly cubic and pyramidal morphologies, respectively, for all the analyzed charges. On the other hand, samples deposited at −0.10 V started growing as pyramids and evolved to cubes with the increase of electrodeposited charge, showing the dependency of the film growth on the thickness. In this case, the change in the growth orientation could have been induced by the emergence of the rhombohedral phase observed in Figure 2 for −0.10 V. In the cross-section images, column 4, films grown at 0.30, 0.10 and −0.10 V and the same growth charge of 50 mC have thickness of 1000, 500 and 800 nm, respectively. A compact and columnar layer is observed, with larger grains on the top, especially for samples grown at 0.30 V.

**Figure 4:**
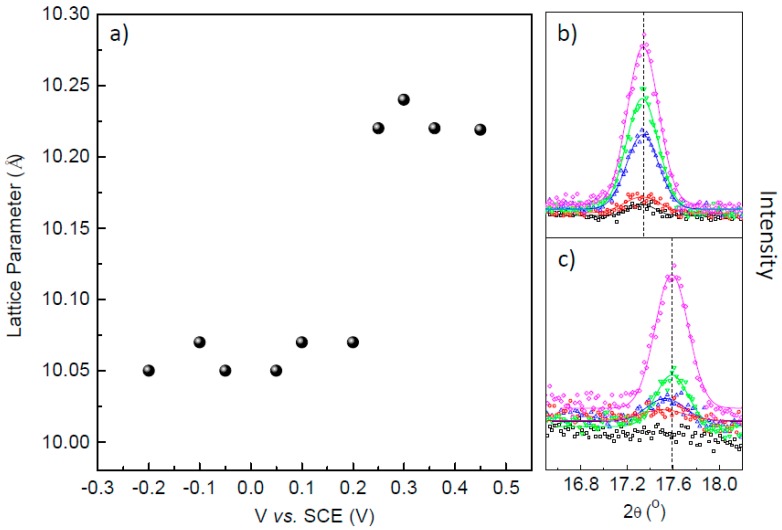
(**a**) The lattice parameter as a function of deposition potential. The amplified (200) peak for samples deposited at (**b**) 0.30 V and (**c**) −0.10 V for electrodeposited charges of 10, 20, 30, 40 and 50 mC fitted with Gaussian curves.

Figure 4a shows the lattice parameter (a) as a function of deposition potential. The values were obtained throughout XRD spectra by analyzing the (200) peaks. A clear step can be seen at 0.20 V that defines the transition from PB (a ~ 10.23 Å) to PW (a ~ 10.06 Å). The incorporation of K^+^ ions to maintain the charge neutrality reduces the lattice parameter of the atomic structure. The observed transition voltage is well defined by the XRD data and is in agreement with results from current transients, thickness and Raman presented above in Figure 1 and Figure 2. Figure 4b and 4c shows the (200) XRD peak for samples prepared at 0.30 and −0.10 V for electrodeposited charges of 10, 20, 30, 40 and 50 mC, i.e., for increasing thicknesses. Both sample sequences have a preferential growth at [200] orientation. As expected, higher peak intensities are seen for thicker samples. For the sample deposited at 0.30 V, Figure 4b, one can see that the electrodeposited charge has no influence on the lattice parameter. However, for the films deposited at −0.10 V, Figure 4c, higher values of the lattice parameter can be inferred for the thin layers.

**Figure 5:**
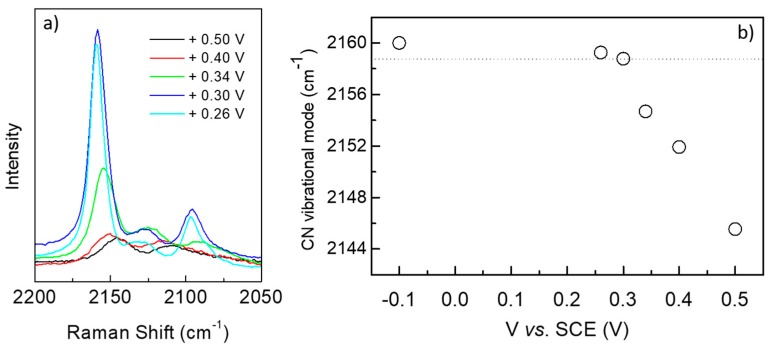
(**a**) Intensity and (**b**) peak position of the Raman peak for the existence of Fe^3+^ species in the layers as a function of deposition potential. Electrodeposited charge for growing the layers was 50 mC.

Figure 5a shows a sequence of five Raman spectra of samples prepared with the applied voltage decreasing from 0.50 to 0.26 V. The intensity of the peak at higher wavenumbers, characteristic of PB phase, increases significantly for potentials close to the lower limit of this interval, i.e., in the region where the maximum thickness was observed (see Figure 1c). At the same time, a linear increase in the wavenumber of this peak from 2144 up to 2160 cm^−1^ is observed, as plotted in Figure 5b. In this figure, the stable value of 2160 cm^−1^ is also observed for depositions at -0.10 V, a voltage that is typical for PW growth. The wavenumbers lower than 2160 cm^−1^ are probably due to the formation of defective PB layers associated with the decrease in the deposition efficiency observed in Figure 1c for potentials above 0.30 V and also taking into account that the CN stretching band is sensitive to variations in: (i) oxidation state of the coordinating metal, (ii) atomic structure and (iii) coordination number [44,45]. From these Raman data and the thickness plot of Figure 1c, we could define a deposition voltage interval, from 0.30 to 0.22 V, suitable for the growth of PB deposits. The vibration peak at 2160 cm^−1^ that appears in the spectrum of film grown at −0.1 V (see Figure 2 and Figure 5b) would be an indication of the existence of Fe^3+^ species in these layers, an unexpected fact for this deposition potential that needs further investigation.

## 4. Conclusions

Thick, compact and uniform layers of Prussian Blue and Prussian White were obtained with morphology and structure that are dependent on the deposition potential and thickness, as demonstrated by SEM images, X-Ray diffractograms, and Raman spectra. A sharp transition was observed in the applied voltages for growing PB and PW, delimitating the regions for electrodeposition of these materials. A restricted voltage interval from 0.30 to 0.22 V was considered as adequate for growing PB layers with high quality and efficiency. Preferential growth in the <111>, <200> and <311> directions was observed, with the formation of cubes and pyramids on the surface, that was strongly dependent on the deposition potential. For voltages of 0.00 and −0.10 V, a mixture of the cubic and rhombohedral phase was revealed by the diffractograms. In summary, by controlling deposition potential and thickness, distinct layers of PB and PW were prepared for potential application in devices such as supercapacitors and batteries.
